# Supplementary material for: Trend and decomposition analysis of factors influencing teenage pregnancy and motherhood in Nigeria, 2003–2018
Source: PLoS One. 2025 Jun 11;20(6):e0325659. doi: 10.1371/journal.pone.0325659 (PMC12157668; doi:10.1371/journal.pone.0325659)
Supplement: Attachment — Submitted filename: Point-by-point response.pdf [file pone.0325659.s002.pdf]

April 18, 2025

Dear Editor,

Cover Letter: PLOS ONE Decision: Revision required [PONE-D-24-38929] - [EMID:6d4f8c277d46a2b2]

Thank you for the review of our paper titled "**Trend and decomposition analysis of factors influencing teenage pregnancy and motherhood in Nigeria, 2003 – 2018**". The comments are valuable in terms of enriching the quality of the paper.

Please find the responses to the editorial and reviewer's comments below for your information and attention.
Regards!

Yours sincerely,

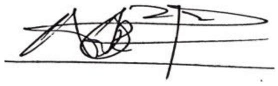

Dr Rotimi Afolabi
rotimifelix@yahoo.com
On behalf of the authors

Reviewers Notes and Responses

S/N	Comments	Authors’ responses and Amendment Made
I	**Editorial comment - ACADEMIC EDITOR**	
	The manuscript provides a well-structured analysis of trends and factors associated with teenage pregnancy and motherhood (TPM) in Nigeria over 15 years. In addition to expert reveweers' feedback, below are some comments for the authors to consider:<br>• Ensure consistency in terminology<br><br>• Ensure sentences make complete sense. For example, ‘This has contributed …’ (line 66). What does ‘this’ refer to? Please, extend this correction to all relevant sections of the manuscript.<br><br>• ‘Research has revealed that achieve a reduction in the global population growth rate …’ (lines 90 – 91), is not clear.<br><br>• Some sentences are lengthy and could be restructured for clarity.<br><br>• Avoid the use of emotive words e.g. ‘serious’<br><br>• Where appropriate, use ‘childbirth’ instead of ‘delivery’	We appreciate these comments. The comments have been addressed in the revised manuscript.<br><br>• The comment is well received. We have ensured consistency of terminology in the revised version of the manuscript.<br><br>• The sentence has been modified and we have extended similar corrections to all relevant sections as suggested. Please refer to<br>○ **Introduction, lines 66-77, 113**<br>○ **Methods, lines 206, 224, 227**<br>for the detailed modification in the revised manuscript<br><br>• The sentence has been modified for clarity’s sake. The statement now reads as “Empirical evidence has shown that Nigeria is among the countries identified for targeted interventions to reduce the global population growth rate”. Please refer to<br>○ **Introduction, lines 97-99.**<br>for the detailed modification in the revised manuscript<br><br>• The lengthy sentences have been shortened and restructured for clarity, as suggested. Please see the revised version of the manuscript<br><br>• The word “serious” has been deleted and the sentence revised accordingly as suggested.<br><br>• The word “childbirth” has been replaced with the word “delivery” where appropriate, as suggested. Kindly refer to<br>○ **Introduction, lines 76 & 77**

		for the detailed modification in the revised manuscript
	• How did previous studies underestimate TPM in Nigeria? It would be helpful to briefly highlight why previous cross-sectional studies may have underestimated TPM burden in Nigeria.	• The comment is quite appreciated! As suggested, we have included the following statements “This approach fails to account for individuals who experienced TPM in their teens but are now adults, potentially overlooking the true extent of the issue and undermining effective intervention strategies” and “The present study focused on women who have completed their teenage years, considering all pregnancy endpoints - childbirth, miscarriage, or abortion, …” Kindly refer to<br>o **Introduction, lines 103-105 & 110-112**<br>for the detailed modification in the revised manuscript
	• Line 101: how will the study contribute to attaining SDG 3?	• We have provided statements “It could also provide information for adolescent health care services, and for preventing morbidity and mortality associated with TPM, guiding efforts towards achieving the Sustainable Development Goals in Nigeria and worldwide” to address this important concern. Please see<br>o **Introduction, lines 115-117**<br>for the detailed modification in the revised manuscript
	• A brief explanation of why the decomposition method is particularly suited for TPM analysis would strengthen the rationale, especially, in comparison to previous studies.	• This comment is appreciated. We have included the following statements “Additionally, studies have assessed trends and investigated factors associated with teen pregnancy and childbearing. However, little is known about the changes in TPM and the drivers of these changes over time in Nigeria.” & “…and employed multivariate decomposition analysis (MDA) to account for the changes in TPM across the studied period.” in the revised version of the manuscript. Kindly see<br>o **Introduction, lines 106-108 & 112-113**<br>for the detailed modification in the revised manuscript
	• The manuscript highlights important intervention points, but it would benefit from more specific policy recommendations.	• The suggestion is equally appreciated. Specific policy recommendation has been included in the revised version of the manuscript. Please see<br>o **Conclusion, lines 463-464**

		Again, thank you for all the comments, corrections and suggestions to ensuring the manuscript is improved.
II	**Editorial comment - Journal Requirements**	
	1. Please ensure that your manuscript meets PLOS ONE's style requirements, including those for file naming. The PLOS ONE style templates can be found at https://journals.plos.org/plosone/s/file?id=wjVg/PLOSOne_formatting_sample_main_body.pdf and https://journals.plos.org/plosone/s/file?id=ba62/PLOSOne_formatting_sample_title_authors_affiliations.pdf.	Th comment has been addressed in the revised manuscript by including "Nigeria" to the first affiliation. Please refer to:<br>○ **Title page, line 8**
III	**Reviewer #1: Comments**	
1.	The paper provides the valuable insights into the dynamics of teenage pregnancy in Nigeria and also methodologically sound. in order to improve the manuscript further, the authors should address the following points<br>1. Teenage pregnancy and motherhood (TPM) should be operationally defined<br>2. Authors should acknowledge the limitations related to self-reported data and cross sectional nature of the study design.	Thank you for this comment and recommendation. In line with the reviewer observations, we have addressed them in the revised version<br>1. This comment is appreciated. We have provided the statement "" to operationalised TPM in the revised manuscript. Please see<br>○ **Introduction, lines 55-57**<br>2. We equally thank the reviewer for this comment. Limitations related to self-reported data and cross-sectional nature of the study design were included in the limitations section earlier. Please refer to the revised version of the manuscript.<br>○ **Limitations, lines 444-447**<br>Again, the comments are appreciated. Thank you!
IV	**Reviewer #2: Comments**	
2.	• Add inclusion and exclusion criteria details, why age range is till 49, what about abouve that age.	• This comment is well appreciated. The parent study - DHS, considered women of reproductive age, aged 15 to 49 years. The present study followed the pregnancy history of adults during their teenage years to estimate TPM levels. Thus, the study included only women who had attained age 20 years and above, and reported at least one childbirth, stillbirth or pregnancy termination including all required dates for relevant events as of the survey date. In other words, women age 19 and below are excluded from the study. Please refer to

		○ **Methods, Study population subsection, lines 140-144**<br>○ **Methods, Study variables, Outcome variable subsection, lines 153-154**<br>for the detailed modification in the revised manuscript
	• Also, authours can add acxess to health case dacility or primary health education, role of family planning services in that area, it should be discussed or assesed through data.<br>• Highly appreciated the aim of the study to assess the trends and was also conpare to some extend but what is the outcome, what is next, what is the importance of this trend data, will it be compare with governemnt interventions ?, authors can further add the cimparision disscusion between two stat3s where one has highest numbers of TPM and other has reducing trends in terms of elaborating what majors that other srate has raken to reduce the numbers.<br>• Elaborate the aim in the abstract so that readers can get the main important goal of this study.	• We quite appreciate the reviewer's comment in this regard. In line with this important observation, statements were provided to address this concern in the revised manuscript. Please refer to<br>**Discussion section, lines 380-388**<br>• We appreciate the comments and recommendations. The study focus did not include the subnational analysis. However, we plan to implement another study that will consider comparison between states with highest numbers of TPM and other states with a reduced TPM as suggested.<br>Thank you for this important observation<br>• This comment is appreciated. Meanwhile, authors are constraint by the words count limitation in the abstract. Nonetheless, the aim has been modified in the revised manuscript. Please see<br>• **Abstract, lines 27-28**<br>The comments and suggestions are greatly appreciated. Thank you
V	**Reviewer #3: Comments**	
3.	The paper is overall inerenting, however some comments are necessary:<br>- In the Abstract I would rather speak of "the limited number of studies"	We appreciate the reviewer's recommendation. We have addressed all the comments in the revised version of the manuscript.<br>We appreciate this correction! The sentence has been modified accordingly in the revised manuscript. Please refer to

	- lines 105-115 all goes to introduction, two lines maximum can be kept to provide the specific context of the study maybe also changing the paragraph title to include the word "context" although it is not mandatory<br><br>- lines 120-1212 eliminate this unnecessary phrase<br><br>- In general, the English form needs revising, eliminate spelling mistakes like 20013, 247 and other lines, please choose better vocabulary for scientific purposes.<br><br>- Please clarify in the methods why the specific focus on women reporting and reported at least one childbirth, pregnancy termination, or 32 stillbirth before attaining age 20 and not just considering all women experiencing pregnancy in that age class, considering you cite in line 143 "irrespective of whether the pregnancy resulted in childbirth or was voluntarily or involuntarily terminated". This is a major issue	○ **Abstract, lines 26-27**<br><br>We share the reviewer's concern. The subheading has been modified to read as "Study area" and some statements moved to introduction as suggested in the revised manuscript. Please refer to<br>○ **Methods, Study area subsection, lines 120-124**<br><br>We thank the reviewer for this comment. We have expunged unnecessary phrases and modified accordingly in the revised version of the manuscript.<br><br>This very important suggestion by the reviewer is appreciated. We have edited its grammar and eliminated spelling mistakes accordingly, and we hope that all grammar issues have been well addressed.<br><br>We equally share the concern of the reviewer. Nonetheless, DHS does not directly provide information on teenage pregnancy and motherhood. To generate the TPM indicator variable, we use information derived from the responses to these three questions: "age of respondent at first childbirth", and "have you ever had a pregnancy that was miscarriage, aborted, or ended in a stillbirth?" and "when did the last such pregnancy end?" The prevalence of pregnancies and childbirths among girls within the teen age group is an indicator used in this study to define the magnitude of teenage pregnancy and motherhood. The indicator is retrospective in asking women aged 20 and above (who are no longer teenagers at that time) about pregnancies and childbirth, while they were teenagers. Meanwhile, indicators such as the prevalence of teenagers aged 13 to 19 who are currently pregnant or who have had a live birth is affected by censoring – teens not pregnant or without a live birth still face the risk of pregnancy and/or childbirth before they reach age 19. (Loaiza & Liang 2013).<br>The statement "These studies fail to account for those who experienced TPM as teenagers but are now adults, thereby underestimating its true burden" has been provided in the revised version of the manuscript. Please refer to

	- State clearly in the methods the age class as there seems to be inconsistency between abstract and manuscript methods. Furthermore, detail on age class needs to go the results section<br><br>- You mention “The detail of the ethical approval was reported by Measure DHS”. Please state exactly where it was reported and a reference to find this approval<br><br>- The Discussion section is too long, please sum up and leave out inessential concepts<br><br>- With regards to lines 315-320, a major role is also played by public health policies and programs in place. Please expand on different public health approaches of these countries so as to add complexity to the understanding of observed differences among these countries	○ **Methods, Study variables, Outcome variable subsection, lines 151-152**<br><br>The analysed data consist of women who were age 20 and above, who had completed their teen years but had history of pregnancy, childbirth, miscarriage or termination of pregnancy as a teen, i.e., before age 20 years. The clarity’s sake, the lowest category of the age group (in years) has been replaced with 20-24 in the revised version of the manuscript, including all the Tables.<br><br>This important observation is appreciated. References on where the ethical approval has been reported have been included in the revised version of the manuscript. Kindly refer to<br>○ **Methods, Ethical approval subsection, line 239**<br><br>The length of discussion section has been reduced by expunging some statements with inessential concepts as suggested. Please refer to Discussion section for the modified version of the manuscript<br><br>This comment is equally appreciated. We have included the phrase “… such as exposure to media, socioeconomic factors, and family dynamics …” in the revised version of the manuscript. We hope the modified version meet the reviewer expectation as we are equally considering reducing the length of the Discussion as early commented. Please refer to<br>○ **Discussion, lines 336-337**
VI	**Reviewer #4: Comments**	
1	1. The study offers valuable insights into the trends and determinants of teenage pregnancy and motherhood in Nigeria. There are some comments to improve your manuscript:<br>1. What is the meaning of "media exposure"?	This very important observation is highly appreciated. We have addressed these important comments in the revised version of the manuscript.<br><br>1. Media exposure according to DHS refers to women who are exposed to media (specifically, reads newspaper, watches television, and listens to radio) on a weekly basis. Thus, having access to at least radio, television, or

		newspaper was categorised as "exposed", otherwise, "none". Please refer to<br>• **Methods, Study variables, Explanatory variables subsection, lines 178-179** for the detailed modification in the revised manuscript
	2. How was household wealth status measure?	2. The household wealth status variable, derived from the generated weighted factor score by principal component analysis as contained in the women recode file, was grouped into poor, average, and rich wealth tertiles. It is a proxy measure of household socio-economic status due to the non-existence of information on household income. The factor scores were generated based on ownership of household assets (e.g., television, radio, or bicycle/car) and housing characteristics (e.g., flooring, wall or roofing materials) using principal component analysis. For the detailed modification in the revised manuscript, please see<br>• **Methods, Study variables, Explanatory variables subsection, lines 179-182**
	3. The lines 173-176 are not clear. The method used to define community SES needs more detailed explanation.	3. For clarity's sake, the detailed explanation on how community SES disadvantaged index was derived has been provided in the revise version of the manuscript. Kindly refer to<br>• **Methods, Study variables, Explanatory variables subsection, lines 182-192**
	4. How was the test for asterisk number in table 2 conducted? Please mention the name of the test and explain the difference between the tests with the reported p-values on the last column of Table 2.	4. The chi-square test for trends of proportions was used to identify the significant changes over the studied period (as stated in **Methods, Statistical methods of analysis subsection, lines 200-201).** The associated p-values of the test were reported as the last column.<br>The asterisk was only meant to highlight the significant upward (increasing) trend, while the significant downward trends were NOT asterisk. This is included as part of **Table 2 footnotes**.
	5. Some percentage in lines 290-302 do not exactly match the percentage in Table 3. This may be due to rounding. However, it is suggested that the percentage should be equal to those in Table 3. For example, report 134.7% instead of 134.7% or 5.6 % instead of 5.7%.	5. Yes, the differences in values are as a result of rounding off to 1 decimal place. We have retained the 1dp for consistency's sake and make the necessary corrections in the body of the text. Please see **Table 3** for the necessary modifications in the revised manuscript

	6. In Table 3, please report the standard error of the coefficient or the 95% confidence intervals.	6. Additionally, we have included the standard error as the inclusion of the confidence interval could make the Table too clumsy as a result of 4dps. Please see **Table 3** for the necessary modifications in the revised manuscript.<br><br>We thank the reviewer for all these insightful comments!
VII	**Additional corrections based on authors' consideration**	1. Level 2 heading tagged "Study population" has been created for the study population paragraph in the revised manuscript. Please refer to<br>• **Methods, Study population, lines 138-144**<br>for the detailed modification in the revised manuscript<br><br>2. Timing of marriage variable's categories have been revised as: unmarried at 20, early-teen (< 15 years), mid-teen (15 – 17 years), and late-teen (18 – 19 years) in the revised version of the manuscript<br><br>3. We also removed the duplicated references in the revised manuscript<br><br>**Thank you.**
